# Regressing grasping using force myography: an exploratory study

**DOI:** 10.1186/s12938-018-0593-2

**Published:** 2018-10-23

**Authors:** Rana Sadeghi Chegani, Carlo Menon

**Affiliations:** Menrva Research Group, School of Mechatronic Systems Engineering and Engineering Science, Simon Fraser University, 250-13450-102 Avenue, Surrey, BC V3T 0A3 Canada

**Keywords:** Force myography, Random forest, Finger movement prediction, Continuous grasping predication, Partial hand prosthesis

## Abstract

**Background:**

Partial hand amputation forms more than 90% of all upper limb amputations. This amputation has a notable effect on the amputee’s life. To improve the quality of life for partial hand amputees different prosthesis options, including externally-powered prosthesis, have been investigated. The focus of this work is to explore force myography (FMG) as a technique for regressing grasping movement accompanied by wrist position variations. This study can lay the groundwork for a future investigation of FMG as a technique for controlling externally-powered prostheses continuously.

**Methods:**

Ten able-bodied participants performed three hand movements while their wrist was fixed in one of six predefined positions. The angle between Thumb and Index finger ($\theta _{TI}$), and Thumb and Middle finger ($\theta _{TM}$) were calculated as measures of grasping movements. Two approaches were examined for estimating each angle: (i) one regression model, trained on data from all wrist positions and hand movements; (ii) a classifier that identified the wrist position followed by a separate regression model for each wrist position. The possibility of training the system using a limited number of wrist positions and testing it on all positions was also investigated.

**Results:**

The first approach had a correlation of determination ($R^2$) of 0.871 for $\theta _{TI}$ and $R^2_{\theta _{TM}} = 0.941$. Using the second approach $R^2_{\theta _{TI}}=0.874$ and $R^2_{\theta _{TM}}=0.942$ were obtained. The first approach is over two times faster than the second approach while having similar performance; thus the first approach was selected to investigate the effect of the wrist position variations. Training with 6 or 5 wrist positions yielded results which were not statistically significant. A statistically significant decrease in performance resulted when less than five wrist positions were used for training.

**Conclusions:**

The results indicate the potential of FMG to regress grasping movement, accompanied by wrist position variations, with a regression model for each angle. Also, it is necessary to include more than one wrist position in the training phase.

**Electronic supplementary material:**

The online version of this article (10.1186/s12938-018-0593-2) contains supplementary material, which is available to authorized users.

## Background

3.6 million individuals are expected to live with an amputation by 2050 [1]. Historical data show that approximately 35% of amputations are related to upper extremities and the majority of these (i.e. over 90%) are partial hand amputation [1]. Partial hand amputation (amputation of digits or hands), which is also known as minor upper-limb loss, can impose a notable influence on the person’s life. It can have an adverse effect on their self-image, can cause loss of job and emotional distress [2]. Epidemiological studies show that the number of partial hand amputations per year is one every 18,000–20,000 residents [3]. A 2018 report indicates that there were 209,053 hospital visits specifically related to hand and digit injuries between 2002 and 2010, and 5000 work-related finger amputations in 2010 in the United States alone [4]. Although the minor amputation is a common injury, due to the vulnerability of the fingers, the research in the field of partial hand amputation has moderately progressed compared to the research in major upper limb amputation filed [3, 5]. One solution to resolve some of the problems regarding the amputation is to fit partial hand amputees with a prosthesis [3]. Several prosthesis options have been investigated and marketed by researchers and companies. Some of these options include passive, body-powered, activity-specific, and externally-powered prostheses [6]. The externally-powered prostheses use myosignals (signal from muscles activation and function) from the user to predict their intention to control the hand. In the commercially available externally-powered prosthesis, surface electromyography (sEMG) is more frequently used due to its portability and non-invasiveness.^1^.

The commercial prostheses provide a set of pre-programmed hand gestures for the user. With correct sensor placement and training, the user can control the hand to shape one of the pre-programmed hand gestures. Although this classification approach can achieve a high classification accuracy, it limits the user to some specific hand gestures [7].

The user of an externally powered prosthesis will not be limited to a set of hand gestures if they can control each finger continuously. To provide such control for the users, researchers have investigated different regression methods to predict continuous finger movements and grasping actions using sEMG [8–10]. The majority of the researchers asked the participant to keep their hand in a specific position and did not consider the effect of wrist movement on the performance of their technique. As a result, Pan et al. [11] collected data in different wrist positions from the Middle finger of six able-bodied participants and the Index finger of two partial-hand amputees. The result of their study showed the possibility of predicting the finger movement in the presence of wrist movement with sEMG signal.

Although the sEMG signal is a common signal to control prostheses, it has some disadvantages. The sEMG signal can be unstable due to the environmental factors, like electrical noise, and user-based factors, such as sweating. This disadvantages resulted in marginal progress despite extensive research on the signal [12]. A low-cost alternative to sEMG to control a prosthesis is FMG. FMG is defined as tracking the volumetric changes in a muscle associated with the muscles contraction or relaxation during the functional movement of the limb [13]. The FMG technique presents some advantages over sEMG technique. Unlike the sEMG technique the FMG technique does not need skin preparation; the signal is not sensitive to the users sweating; and the electrical noise does not affect the signal [14]. Researchers have investigated FMG for hand gesture classification and continuous force prediction during the past decade [13, 15–18]. In two separate researches Connan et al. [19] and Jiang et al. [18] studied the FMG and sEMG signals that were collected from the participants at the same time while they were holding specific wrist positions or hand gesture. They have shown that FMG has a comparable performance to sEMG, during hand and wrist movement classification.

Cho et al. [20] used FMG signal collected from the residual and intact limb of transradial amputees to classify different hand gestures. They compare the classification accuracy between the signal collected from the residual limb and the intact limb. Their investigation indicate that the residual limb has a lower accuracy during the classification, due to the degraded muscle tone. However, they were able to classify six hand gestures with good accuracy in the residual limb. The work of Cho et al. [20] along with other works [21, 22], have showed FMG has the potential to yield promising result when used to control a prosthetic arm in transradial amputee. During the experiment Cho et al. [20] asked the participants hold their elbow in a 90° angle. To investigate the possibility of resolving this constrain, Radmand et al. [17] introduced a high density FMG. They were able to classify hand and wrist motions with a low classification error. The authors also suggested to include data from eight different static positions in the training dataset. This positions were defined as specific locations in 3D space in front of the participant to cover a person’s workspace and ensure proper performance with limb position variation. Using different static positions resulted in classifying hand and wrist gestures in 3D space with a high classification accuracy.

As FMG signal demonstrates promising results for hand and wrist classification, Kadkhodayan et al. [23] used the signal for continuous finger movement prediction. They recorded the relative displacement of the tip of the Index finger, the Middle finger and the Thumb with respect to a reference point on the hand, during three hand movements. The authors asked the participants to keep their hand in a strictly fixed position. They used a support vector regression (SVR) with a Radial bases kernel function as their regression algorithm. The authors used ten-fold cross-validation to validate the model’s performance. The work of Kadkhodayan et al. [23] can indicate the potential use of FMG signal for continuous grasping movement prediction.

One point about the work of Kadkhodayan et al. [23] is worth mentioning. Kadkhodayan et al. [23] asked participants to keep their hand in a stationary position. In partial hand amputees with a functional wrist, the attempt to perform a hand grasp, either with the remaining digits or to control a prosthesis, is accompanied by wrist movement. Pan et al. [11] mentioned since the wrist movement affects the myosignal it has a negative effect on the system’s performance during continuous movement prediction. In other words, the system trained in a fixed hand position may not be able to perform as well with different hand positioning [5]. The same effect might be observed in prediction using FMG signal.

In our study, the performance of FMG for continuous grasping prediction is investigated. To build upon the work of Kadkhodayan et al. [23], we included different wrist positions in the study. To the authors’ best knowledge the effect of the wrist positioning on the FMG performance for predicting continuous grasping movement has not been investigated. In this paper we propose to use random forest (RF) algorithm [24] as our prediction algorithm, due to its potential advantages to the conventional algorithms. Some of the advantages are as follows. The RF algorithm is robust to overfitting; it has only two parameters to optimize (the number of variables in the random subset at each node to split and the number of trees in the forest), while it is not very sensitive to these parameters [24, 25]. The features of the RF algorithm can make it a potential alternative to the conventional algorithms, for the goal of this study. Four regression methods, linear regression (LR), SVR, neural network regression (NNR), RF, and three classification methods, linear discriminate analysis (LDA), support vector machine (SVM), and RF had been investigated and compared in the present work. After determining the model to predict the continuous grasping movement, the effect of the variation of the wrist positioning on the performance of the model was investigated. The investigation was done to determine if it is possible to train on a fixed wrist position and predict the grasping movement in different wrist positions. In addition to that, the minimum number of the wrist positions that need to be included in the training phase was examined. The result of this work can create the groundwork for a further investigation of the potential of the FMG technique to be used as a controlling technique for continuously controlling a prosthesis device.

This paper is organized as follows: next section explains the proposed experimental protocol and processing of the experimental data; after that, an overview of experimental results is provided. Discussion and concluding remarks are presented at the end.

## Methods

This section describes the setup and methods that were used for the data collection. It also specifies the different grasp types and wrist positions that were used during the data collection. In addition to that the processing of the data and the machine learning algorithms are defined. The last part presents the outcome measures to evaluate the performance of the models.

### Experimental setup

**Fig. 1:**
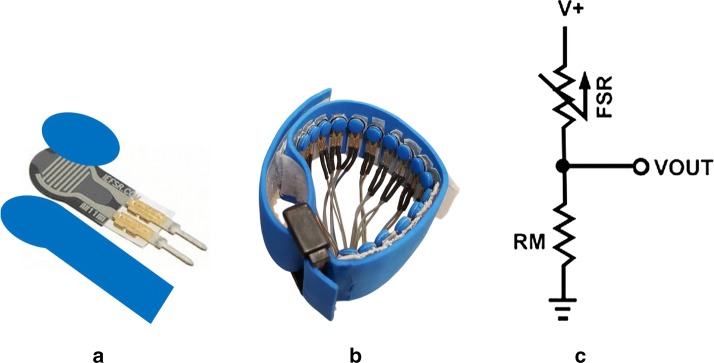
**a** The configuration of the hard backing and the foam on the FSR, **b** the FMG data collection band, **c** the FSR circuitry. VOUT is recorded with the microprocessor

An array of 18 force sensing resistors (FSR^®^ 400, Short, Interlink Electronics, Westlake Village, CA) were placed in a flexible band, cut from 2 mm thick foam. Figure 1a shows a close view of a FSR. FSRs are polymer thick film (PTF) sensors that show a decrease in resistance by increasing the applied force on their surfaces. This specific model can be activated with 0.2 N force, and its sensitivity range is up to 20 N [26]. As the FSRs are flexible devices, by wrapping the band around the participant’s wrist, each FSR will bend. To stop the FSRs from bending, each FSR was supported with a piece of 1.5 mm thick acrylic sheet which was cut to the FSR’s exact shape and dimensions. The acrylic piece provided a hard backing for the FSR which stopped it from bending. A piece of 1.5 mm thick foam was placed on each FSR, to concentrate the pressure from the wrist on the FSR’s sensing area. The sensors were placed on the flexible band, 4 mm apart from each other. Figure 1b illustrates the FMG data collection band. As mentioned in Interlink Electronics [27] to have a simple force reading the sensor was connected to a resistor in a voltage divider circuit (Fig. 1c). The value of the output voltage read from the resistor (VOUT in Fig. 1c) was used as a measure of the applied pressure. An Atmega 32 microprocessor was used to read this value. The microprocessor was connected to an on-site computer with a USB cable, to record the data. A velcro strap and a buckle were used to wrap the band around the participants’ limb. The band placement was kept uniform among the participants. It was placed on the upper limb above the head of Ulna bone. The buckle of the band was kept on the Radius bone. The reasoning behind this placement is that, the muscles and tendons that control hand digits are mostly deep muscles. The nature of FMG is to pick up the effect of limb volume changes, from muscle and tendon movements, on the skin surface. On the forearm, moving from the elbow to the wrist, the digit controlling muscles get closer to the skin surface, and the changes would be more localized for the FMG band. It is worth mentioning that these changes are visible on forearm belly muscle as well, but they are more localized near the wrist. As the changes regarding the digit movements were of great interest in this research this specific placement was selected.

**Fig. 2:**
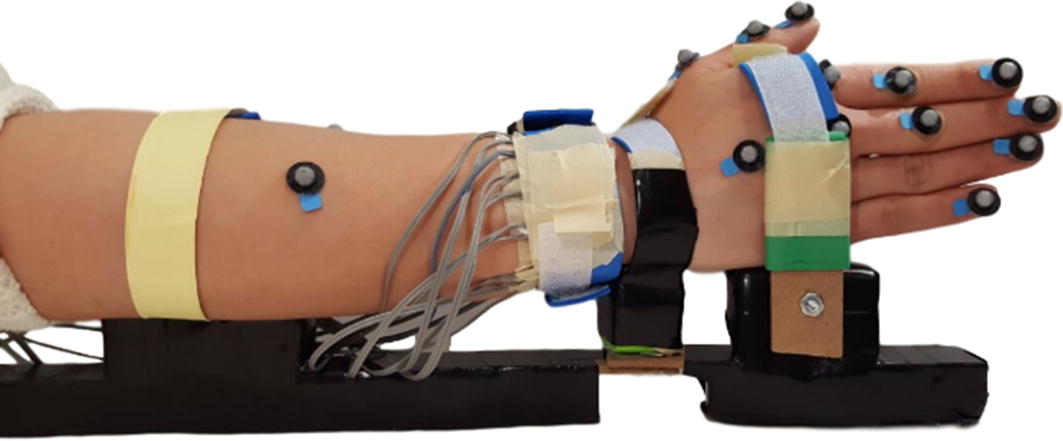
Participant’s hand placement in the brace to eliminate undesirable movement

The participants sat behind a desk with an adjustable height. The height of the desk and their chair was adjusted in a way that participants could rest their elbow on the table. Since the goal of this study was to investigate the effect of the wrist position on the FMG, to eliminate any other unwanted movement, participants’ hand and forearm were fixed in a brace (Fig. 2). To maintain participants’ comfort pieces of the polystyrene foam were used as support for forearm, wrist, and hand in the brace. The brace included a joint under the wrist. For moving to each wrist position the nut and screw of the wrist joint were unlocked. Then the participant was asked to move their hand to the specific position. The nut and screw were locked after hand positioning. With this consideration, the elbow was kept relaxed, and the wrist was fixed in a specific position during each data collection session.

**Fig. 3:**
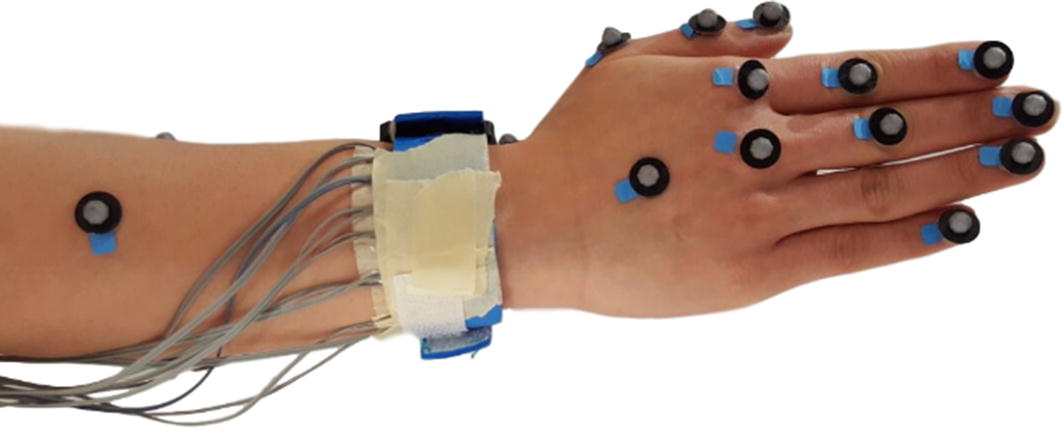
Band and marker placement on the participant’s hand

A motion capture system (Qualisys AB, Gothenburg, Sweden) was used to track the finger movements. The system consisted of eight cameras, 15 markers, and the motion capture software which is called QTM. The markers were placed on the participants’ hand, wrist and forearm (Fig. 3). On the index and the Middle finger, markers were placed on fingertips, proximal interphalangeal (PIP) joint, and metacarpophalangeal (MCP) joint. On the Thumb, the markers were placed on the Thumb’s tip, interphalangeal (IP) joint, and MCP joint. On the wrist, the marker was placed on the Radiocarpal joint. On the forearm, the markers were placed on the Radius bone in line with the wrist marker and between the Ulna and Radius perpendicular to the Radius’s marker. Two markers were placed on the Little and Ring fingers’ tips as well. The data collected from the markers using the cameras was used to create a 3D model of the hand, fingers, and forearm to track their movements.

**Fig. 4:**
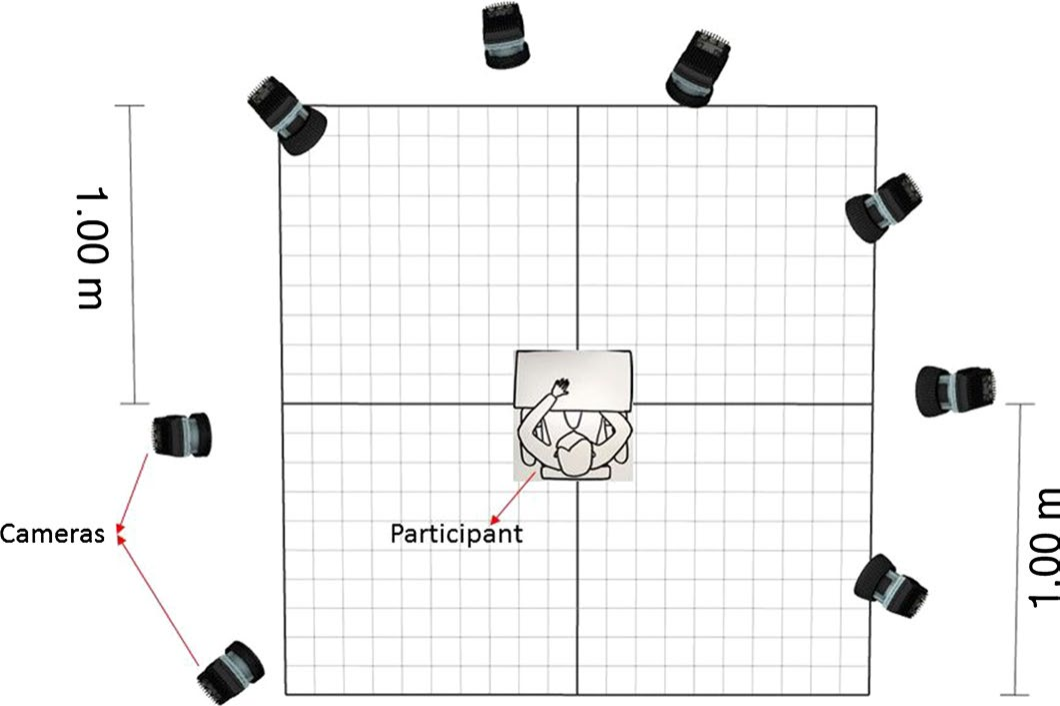
The camera placement around the subject

Each marker on the participant’s hand should be visible to at least three cameras during data collection, for the QTM software to track the position of the marker in 3D space. Figure 4 shows the camera placement and the participant. Cameras were placed around the participant, to cover approximately 250° of the area around them. The camera placement guaranteed that all the markers were at least visible to three cameras, during data collection.

A program in C# was coded, to collect data from the FMG band and the motion capture system at the same time. The program consisted of two threads, main thread and background thread. The band was read in the background thread and the motion capture data was read in the main thread. Every time the program received a data point from the band, the corresponding 3D location data of the markers from the motion capture system was read. The sampling frequency of the motion capture system was set to 100 Hz. The sampling rate of the FMG band was set to 15 Hz. This was sufficient to track finger movements in this study, which is slower than 1 Hz [23].

### Grasp types and wrist positions

**Fig. 5:**
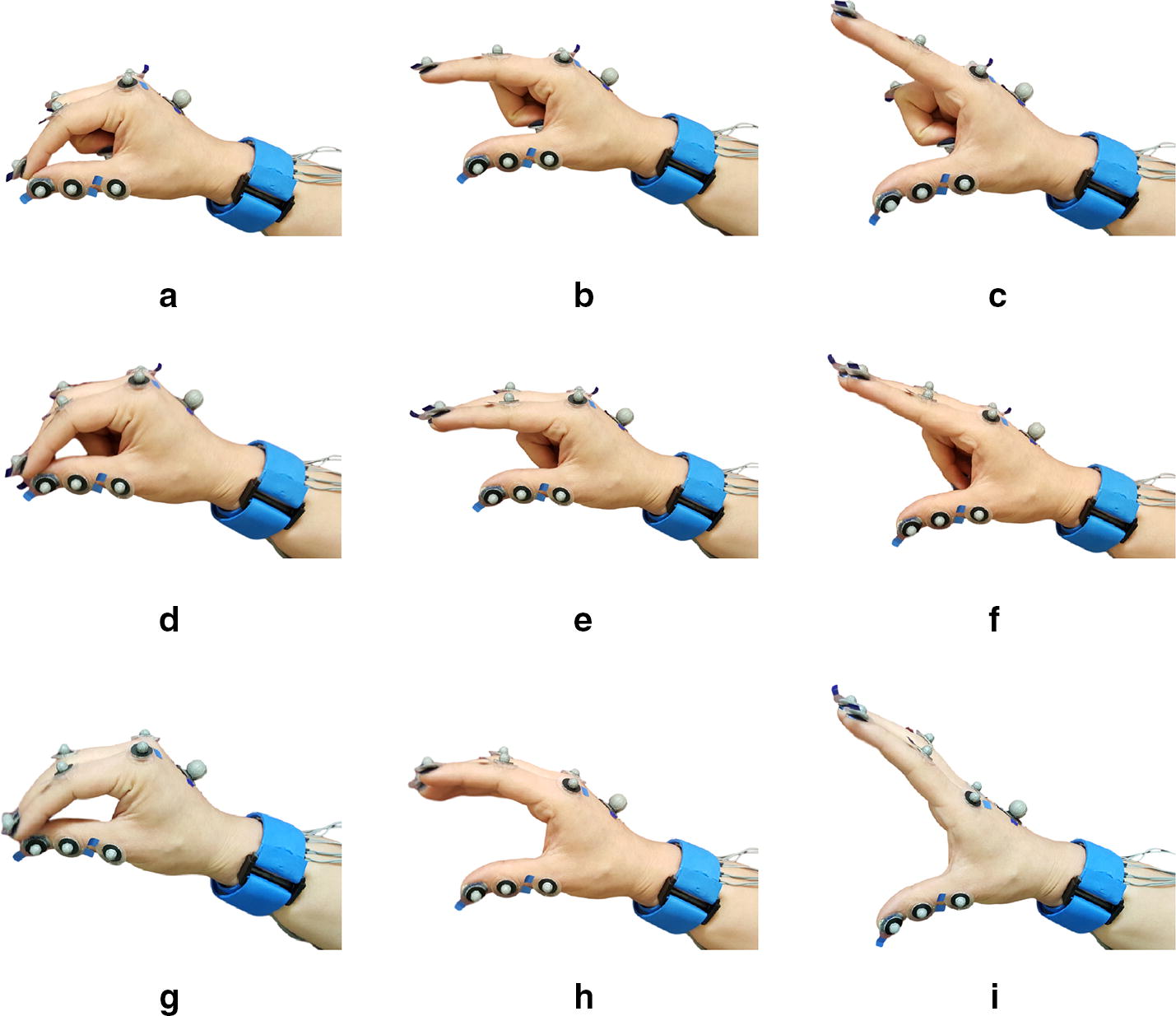
Snap shots of the hand’s motion. **a**–**c** Opposed Thumb-Index Finger grip, **d**–**f** opposed Thumb-Two Finger grip. **g**–**i** Heavy wrap-Large Diameter

Three hand grasps were selected, to investigate the effect of the continuous grasping movements on the FMG. In an attempt to estimate the continuous grasping movement Kadkhodayan et al. [23] selected three grasp types, as a subcategory from Cutkosky’s grasp taxonomy [28]. To build upon the work of Kadkhodayan et al. [23] the same grasp types were selected, and the investigation of the effect of the wrist movement variations was added. The three grasps are opposed Thumb-Index Finger grip, opposed Thumb-two Finger grip, and Heavy wrap-Large Diameter. Figure 5 shows the snapshots of the grasp types during flexing and extending fingers. The first two grasps are precision grasps which mainly are used for manipulating a small object with fingertips. The third grasp is a power grasp and is mainly used for grasping large cylindrical objects. For simplicity the grasps were called “Index Finger-Thumb”, “Two Fingers-Thumb” and “Large Diameter” grasp, for the rest of the paper.

Since three-finger robotic hands and prosthetic hands are commonly used [29–31], the movement of the Index finger, Middle finger, and Thumb were studied in this work. The participants did the grasps repeatedly, which provided data from three dynamic hand movements.

**Fig. 6:**
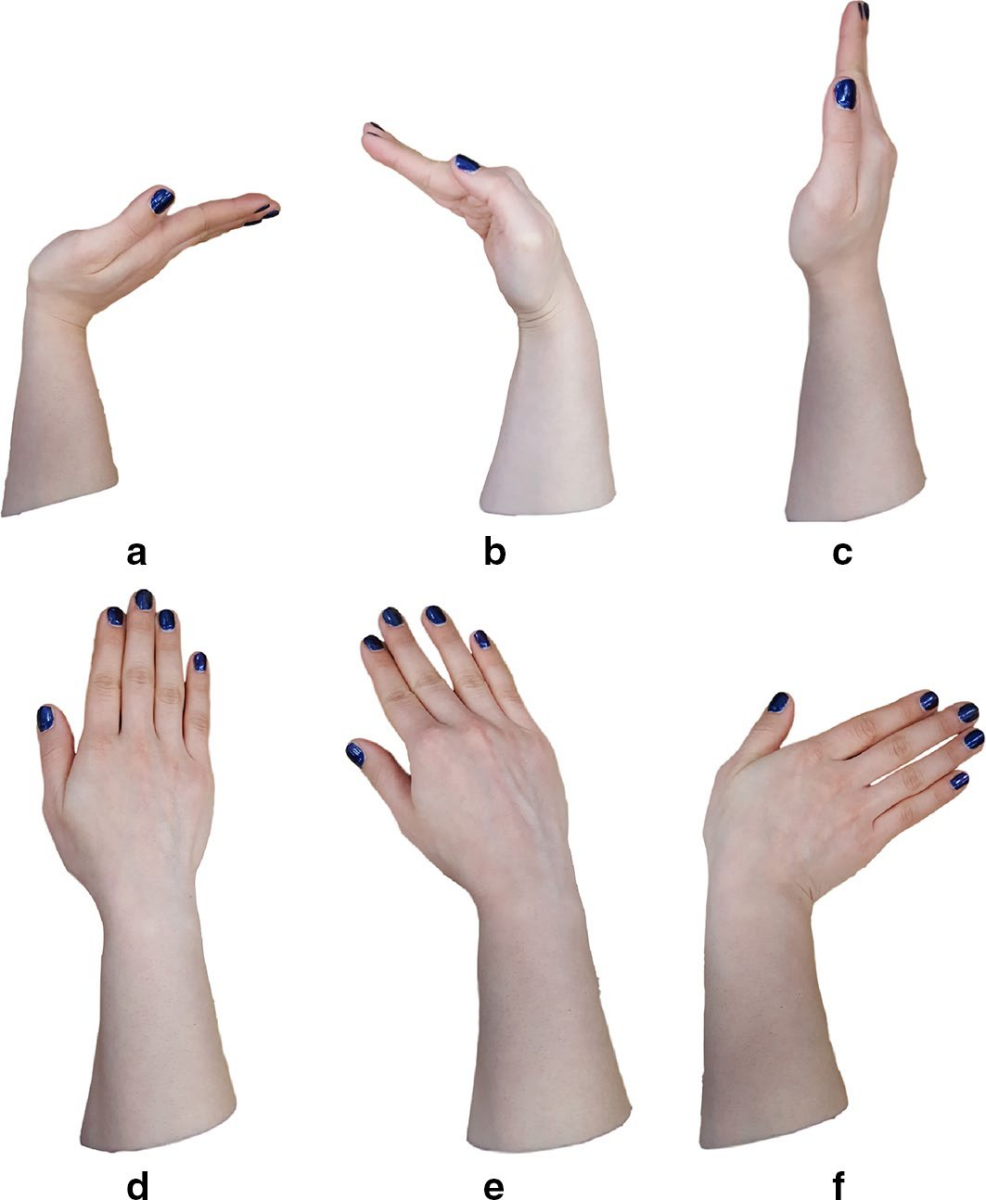
Snap shots of wrist positions. **a** Extension, **b** Flexion, **c** Neutral, **d** Pronation, **e** Radial deviation, **f** Ulnar deviation

In addition to the grasping movement, we looked at the effect of the position of the wrist on the FMG. To do this six static wrist positions were used to collect data. These positions included keeping the wrist in Extension, Flexion, Neutral, Pronation, Radial deviation, and Ulnar deviation. Figure 6 illustrates the wrist positions. This study is a feasibility study regarding the investigation of the effect of the wrist position on the FMG signal, and is a first step toward removing the wrist position effect. Thus, only the six static wrist position following the work of Pan et al. [11] were included in this study, and wrist transition between the static positions was not considered. As the motion capture system was not able to see the markers on the fingers during the hand movements in Supination wrist position, this position was not included in the study. The mentioned grasp types and wrist positions were demonstrated for the participants. After fixing the participant’s elbow and wrist position, they were asked to flex and extend their fingers and Thumb as if they were grasping objects of different sizes. The participants did not hold or squeeze any object. The effect of applying force on an object or surface is not investigated in this study, since it is a feasibility study on the wrist movement effect on the signal. The participants were asked to do each grasp for 10 s. As there are three hand movements this adds up to 30 s in total. The participants repeat the hand movements 30 times. Since the sampling frequency of the band was set to 15 Hz, the repetitions provided approximately 13500 data point for each wrist position. The dataset for each data collection session nearly included 81000 data points. Participants could rest between the repetitions. Participants were asked if they need a rest every 10 min. In addition, there was a mandatory rest half way through the experiment for 15 min. There were no limitations on the speed, and participants performed the motion at their natural pace. With the considerations above, the data collection session for each participant, including setup, rest between repetitions, and recording data, was approximately 3 h long. It is worth mentioning that the fatigue has a lower influence on FMG signal than sEMG signal [19].

**Fig. 7:**
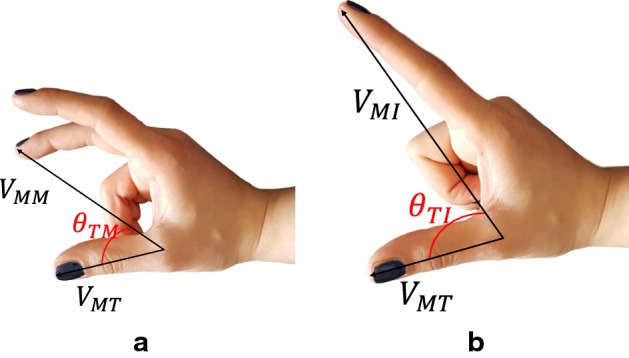
**a** The angle between Middle finger and Thumb, **b** the angle between Index finger and Thumb

In the partial prosthetic hands usually the individual control of each finger joint is not provided, and the hand does not have the same kinematic of the real hand. As a result, if the finger joints are predicted, to control a prosthesis they need to be converted to the kinematics of the prosthesis to be able to control it. This motivated us to select measures different than the finger joint angles for the grasping movement. Kadkhodayan et al. [23] used the length of a vector connecting the fingertips to a reference point on the hand. As this vector can be affected by the size of the participant’s hand, we selected another measure independent of the hand’s dimensions. The angle between Index finger and Thumb and Middle finger and Thumb were selected (Fig. 7).

### The processing of the motion capture data

The collected data from the location of the markers were used to calculate three vectors. The first one was defined as the vector starting from the MCP joint of the Thumb and ending at the Thumb’s tip ($V_{MT}$). The second vector started at the MCP joint of the Thumb and ended at the Index finger’s tip ($V_{MI}$). The last vector started at the MCP joint of the Thumb and ended at the Middle finger’s tip ($V_{MM}$). These vectors were used to calculate the angle between the Index finger and the Thumb ($\theta _{TI}$), and the angle between the Middle finger and the Thumb ($\theta _{TM}$). Figure 7 illustrates the angles. Formulas 1 and 2 show the calculation of each angle.

1 $$\begin{aligned} \theta _{TI}= & {} \tan ^{-1}\left( \frac{|\vec {V}_{MT} \times \vec {V}_{MI}|}{\vec {V}_{MT} . \vec {V}_{MI}}\right) \end{aligned}$$

2 $$\begin{aligned} \theta _{TM}= & {} \tan ^{-1}\left( \frac{|\vec {V}_{MT} \times \vec {V}_{MM}|}{\vec {V}_{MT} . \vec {V}_{MM}}\right) \end{aligned}$$

Considering the six wrist positions and three hand movements, the dataset included 18 different states (3 hand movements in 6 wrist positions) for $\theta _{TI}$. As the Middle finger was not moving in Index Finger-Thumb grasp, the data from this movement was not included in the dataset of $\theta _{TM}$. This provided 12 states (2 hand movements in 6 wrist positions) for the investigation of $\theta _{TM}$.

### Classification and regression

For predicting grasp movements, two approaches were explored and compared. The approaches are one-step regression and two-step regression. The changes in the value of $\theta _{TI}$ and $\theta _{TM}$ were used as the target variable in training and testing phases, and the FMG data was used as the predictor variable. All data processing and analysis were done in an offline setting. The training and testing were performed on a server running Cenots ver. 6.9 equipped with four twelve-core (2 threads per core) Intel(R) Xeon(R) processors (E7-4860 v2 @ 2.60GHz) and 1000 GB RAM. To have a fair comparison between different models and approaches, all programs were run with a single core.

#### One-step regression

In the first approach, one-step regression, a single regression model was used for training the model and testing it, regardless of the hand movement or the position of the wrist. Both training and testing datasets included data from all static wrist positions and dynamic hand movements. Four regression models, namely LR, SVR, NNR, and RF regression were trained, tested and compared to identify which one performs better toward the goal of this paper. To the authors’ best knowledge, RF has not been used on FMG data before. Thus the algorithm will be explained in short.

Random forest is an ensemble of un-pruned trees. Consider a dataset that has *N* data points, and each data point is constructed from *M* features. In the random forest algorithm developed by Breiman [24], to construct each tree a random subset of the samples including $N^\prime$ data points is selected. In a standard tree each node is split based on all *M* features, but in the algorithm developed by Breiman [24], each node is divided using the best guess among a random subset of the features. So instead of using all *M* feature to make a decision at each node, $M^\prime$ features are used to make a decision. The prediction of a new sample is made by aggregating (the majority of votes for classification and averaging for regression) the prediction of the whole forest.

In this paper, the random forest package of Matlab (introduced in R2009a) was used. The number of trees was set to 100. Matlab’s default was used for the number of random features to use for splitting at each node ($M^\prime$), which was the square root of the number of features for classification ($M^\prime = \sqrt{M}$) and one-third of the number of features for regression ($M^\prime = \frac{M}{3}$). Matlab’s default was also used to determine the size of the random subset of the data ($N^\prime$) to construct each tree, which was the same as the training dataset ($N^\prime = N$). In our case, we have 18 sensors which provided 18 channels; thus four random features were used for classification and six random features were used for splitting at each node for regression.

#### Two-step regression

Previous works on sEMG signal have shown that the movement of the wrist degrades the signal and there is a need to define separate models for different wrist positions [5, 11]. Pan et al. [11] indicated when sEMG is used with different models for different wrist positions, it is possible to estimate the continuous finger movements. The two-step regression approach was designed with an inspiration of their work. Comparing this approach and the one-step regression approach can indicate that whether FMG needs different models for different wrist position, similar to sEMG. In addition, by including the two approaches we can roughly compare sEMG and FMG for the same application.

This approach consisted of a six-class classifier and six regression models corresponding to six wrist positions. At the first step, the data points belonging to each wrist position were gathered together. For each group, a regression model was trained. Then a classification model was trained to classify the wrist positions. To test a new data point, first, the classifier found the wrist position that the point was in. Then based on the predicted wrist position the corresponding regression model was selected and used to predict the angle.

Three classification methods were explored, to choose the appropriate classification method for the second approach. As LDA and SVM with a Gaussian kernel have been used in the literature to classify wrist and hand movements using FMG, these two classifiers were tested [14, 18, 32]. In addition to those algorithms RF [24, 25] is introduced for classification using the FMG signal.

#### Analysis of the effect of the wrist position variation

As it was mention in the “Background” section, the movement of the wrist can affect the performance of the prediction. To analyze how the wrist position affects the performance first, one wrist position was included in the training phase, while all six positions were included in the testing set. The result of this part can confirm whether it is necessary to train on different wrist positions or not. One-way ANOVA and Tukey HSD test for post-hoc analysis were used to compare including six wrist positions, and including just one of the wrist positions in the training dataset. After that, the possibility of including less than six positions in the training phase, and the inclusion of all six positions in the testing phase was investigated.

To do the investigation, the data of one or a set of wrist positions were left out of the training dataset, while their data were included in the testing dataset. The reasoning behind it was if for instance, the data in the Neutral wrist position was similar to the data in the Pronation wrist position, it would be possible to include just the data from one of those wrist positions in training data while including both of them in the testing phase.

Five cases were considered. In each case, one, two, three, four or five static wrist positions were removed from the training set, while their data was included in the test dataset. In case of removing two to five wrist positions, all combinations of removing wrist positions from the training data were considered. For instance, in case of removing two wrist positions, 15 combinations needed to be considered. Considering all combinations provides 62 different options in total. The performance of each option was calculated and compared to the performance of the system when all six positions were used for the training phase. One-way ANOVA and Tukey HSD test were used to determine which combinations do not have a statistically significant difference with including all six positions in the training dataset.

In all statistical analysis a confidence interval of 95% was considered ($\alpha = 0.05$). The statistical significance test was run on $\theta _{TI}$ and $\theta _{TM}$ independently. For each angle, the statistic tests were run on two outcome measures, namely $R^{2}$, and the percentage root mean square error ($RMSE\%$). The combinations that did not demonstrate a statistically significant difference with including six wrist positions in the training test were identified for each angle, and each outcome measure. The identified combinations that were overlapped between two angles, and within the two outcome measures, were selected as the combinations that can be used instead of including all wrist positions, without influencing the performance of the prediction.

### Outcome measures

Each of the approaches was investigated separately for $\theta _{TI}$ and $\theta _{TM}$. The collected dataset included thirty repetitions of each hand movement in each wrist position. In this work, a cross-repetition method was used for evaluating the performance. In a cross-repetition method, the data collected from a set of repetitions were left out as the testing dataset and were not used for the model optimization. To shape training and testing datasets, for each approach, six repetitions out of thirty repetitions were randomly selected, without replacement and set out for testing and other twenty-four repetitions were used for training. Which means 20% of the data were used in the testing phase and 80% were used in the training phase. The selection of test data set was made five times, to make sure that each repetition has been in the test set at least once. The result is reported as an average among repetitions and participants. $R^{2}$, $RMSE\%$, the average training time, and the estimated prediction time for a new data point were measured as ways to evaluate the performance of the modeling.

3 $$\begin{aligned} R^{2} = 1-\frac{\sum \limits _{i=1}^N (y_i-y_i\prime )^2}{\sum \limits _{i=1}^N (y_i-\bar{y}_i)^2} \end{aligned}$$

4 $$\begin{aligned} RMSE \% = \frac{\sqrt{\frac{1}{N}\sum \limits _{i=1}^N (y_i-y_i\prime )^2}}{y_{max} - y_{min}} \times 100 \end{aligned}$$

Formula 3 shows the calculation of $R^{2}$, and Formula 4 shows the calculation of $RMSE\%$ value. The expected value is shown with *y*, and $y\prime$ shows the predicted value, ${\bar{y}}$ indicates the average of *y* in the test set and *N* indicates the number of data points in the test set. To measure the estimated prediction time for a new data point, the prediction time for the testing phase was measured and divided by the number of data points in the test dataset.

Concerning the two-step regression approach, it is essential to keep in mind that the evaluation measurements were calculated using the final output, which is the predicted value of $\theta$.

Before training the algorithms, the dataset was scanned. If an angle data point was missing the corresponding FMG data was ignored, and the data point was removed. No other pre-processing was done on the FMG signal, and the raw FMG data was used for classification and regression. Train and test datasets were the same in the one-step regression and two-step regression. The same sets were also used for analyzing the effect of the wrist position variation.

## Results

Ten able-bodied subjects, six males and four females, age 23–41 participated in this study. The experiment received ethics approval from Simon Fraser University, and participants gave their written informed consent. The result of each approach is presented separately. The comparison between two approaches helps to identify the appropriate approach, among two for estimating grasping movement. At last, the effect of the wrist position variation on the performance of the model is investigated.

### One-step regression

Table 1 shows the results for $\theta _{TI}$. The $R_{\theta _{TI}}^2$ values for LR, SVR, NNR and RF were 0.694, 0.868, 0.813 and 0.872 respectively. The $R_{\theta _{TI}}^2$ value indicated that RF performed slightly better than SVR, and NNR, while it significantly outperformed LR. The RF algorithm was about four times faster than SVR and more than 49 times faster than NNR during the training phase. The RF algorithm is also significantly faster than SVR and NNR to predict the output for a new data point.

**Table 1:** Regression algorithms comparison for $\theta _{TI}$, one-step regression approach

	$R^2$	$RMSE\%$	Training time (min)	Estimated prediction time (ms) for each sample point
LR	0.6945 ± 0.07	14.59 ± 1.71	0.011 ± 0.012	0.0006
SVR	0.8680 ± 0.03	9.55 ± 1.07	6.172 ± 0.946	1.097
NNR	0.8129 ± 0.06	11.29 ± 1.51	72.925 ± 7.40	3.730
RF	0.8718 ± 0.03	9.40 ± 1.05	1.487 ± 0.14	0.076

Table 2 indicates the results for $\theta _{TM}$. The $R_{\theta _{TM}}^2$ values for LR, SVR, NNR and RF were 0.873, 0.941, 0.911 and 0.941 respectively. Looking at the result of $\theta _{TM}$ regression, it showed the similarity between SVR and RF, while RF was over two times faster than SVR during the training, and more than seven times faster during testing. RF also performs slightly better than NNR, and the NNR is about 33 times slower than the RF algorithm in both training and testing. The result confirms that the RF algorithm is a good alternative to the other three conventional algorithms for this research.

**Table 2:** Regression algorithms comparison for $\theta _{TM}$, one-step regression

	$R^2$	$RMSE\%$	Training time (min)	Estimated prediction time (ms) for each sample point
LR	0.8734 ± 0.05	8.98 ± 1.27	0.008 ± 0.01	0.0006
SVR	0.9415 ± 0.02	6.12 ± 0.83	2.35 ± 0.45	0.585
NNR	0.9110 ± 0.03	7.53 ± 0.97	32.00 ± 3.38	2.449
RF	0.9411 ± 0.02	6.13 ± 0.84	0.95 ± 0.08	0.076

**Fig. 8:**
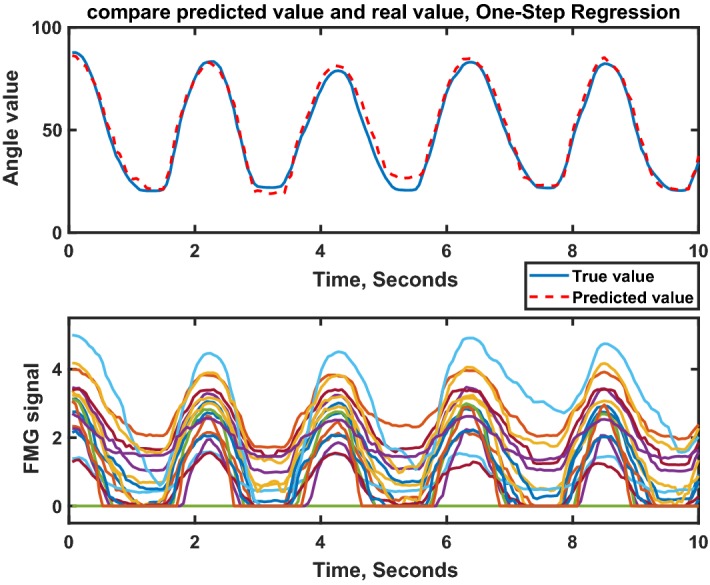
Comparison between predicted value and real value, one-step regression approach using random forest, for $\theta _{TM}$, subject #10, repetition #19, Large Diameter grasp, in Pronation wrist position

Figure 8 illustrates an example of predicting $\theta _{TM}$ using one-step regression approach with RF regression algorithm. Using RF regression $R^2$ value of 0.872 and 0.941 were obtained for $\theta _{TI}$ and $\theta _{TM}$ respectively.

### Two-step regression

**Fig. 9:**
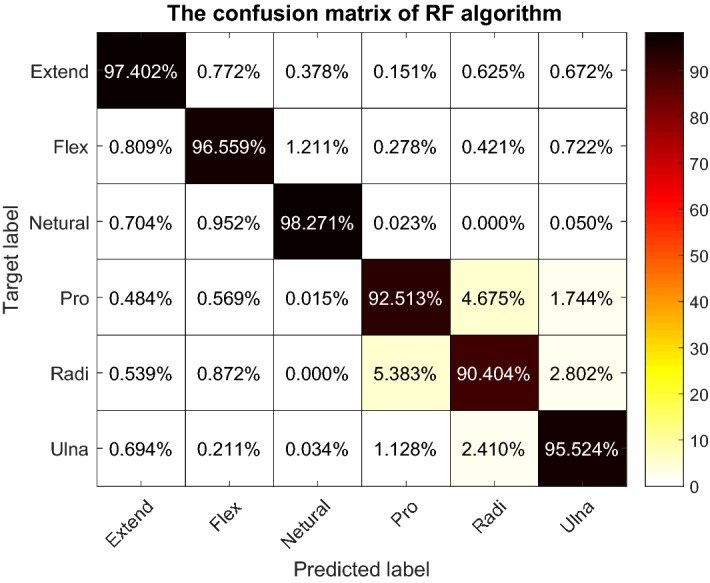
The confusion matrix of RF algorithm during classifying six wrist positions

Based on the result of the first approach, RF was selected as the regression model. Table 3 indicates the result of the classifiers comparison. The accuracy of 85.01%, 95.55%, and 95.76% were obtained for LDA, SVM, and RF on average. RF algorithm was able to do the classification marginally better than SVM, while it was 1.5 times faster, and it outperforms the LDA classifier. The result and training time confirm that RF algorithm can perform as an alternative to LDA and SVM to classify wrist position using FMG signal. Figure 9 illustrates the RF algorithm’s confusion matrix. The confusion matrix indicates that the Pronation and Radial wrist positions are more likely to be miss-classified as each other compared to the other wrist positions. This miss-classification can be a result of the small deviation angle during the Radial deviation, which was an average of 18° for the participants.

**Table 3:** Classification algorithms comparison for classifying six wrist positions

	Accuracy (%)	Sensitivity (%)	Specificity (%)	Time (s)
LDA	85.01 ± 9.55	90.65 ± 11.06	97.87 ± 3.22	0.10 ± 0.07
SVM	95.55 ± 4.41	97.46 ± 4.71	99.52 ± 0.78	58.82 ± 33.26
RF	95.76 ± 4.37	97.42 ± 4.48	99.45 ± 0.92	38.77 ± 3.91

**Fig. 10:**
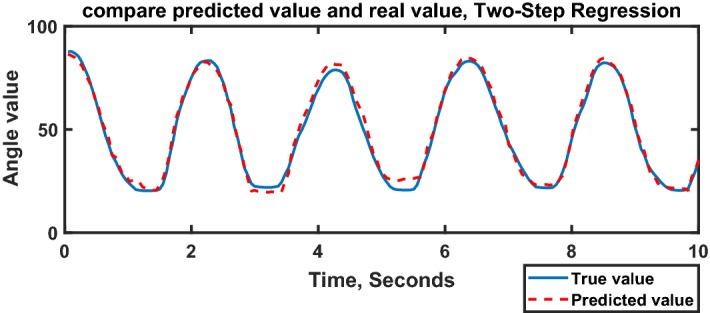
Comparison between predicted value and real value, two-step regression approach using random forest, for $\theta _{TM}$, subject #10, repetition #19, Large Diameter grasp, in Pronation wrist position

Table 4 indicates the average results of the second approach. The $R_{\theta _{TI}}^2 = 0.874$ and $R_{\theta _{TM}}^2 = 0.942$ were obtained. Figure 10 shows a sample of the target and predicted value using the two-step regression approach. The training and testing dataset were the same as Fig. 8.

**Table 4:** The results of two-step regression approach

	Classification accuracy (%)	$R^2$	$RMSE\%$	Training time (min)	Estimated prediction time (ms) for each sample point
$\theta _{TI}$	95.76 ± 4.38	0.8744 ± 0.03	9.31 ± 1.07	2.498 ± 0.179	68.174
$\theta _{TM}$	96.45 ± 3.91	0.9424 ± 0.02	6.06 ± 0.87	1.665 ± 0.117	52.682

**Fig. 11:**
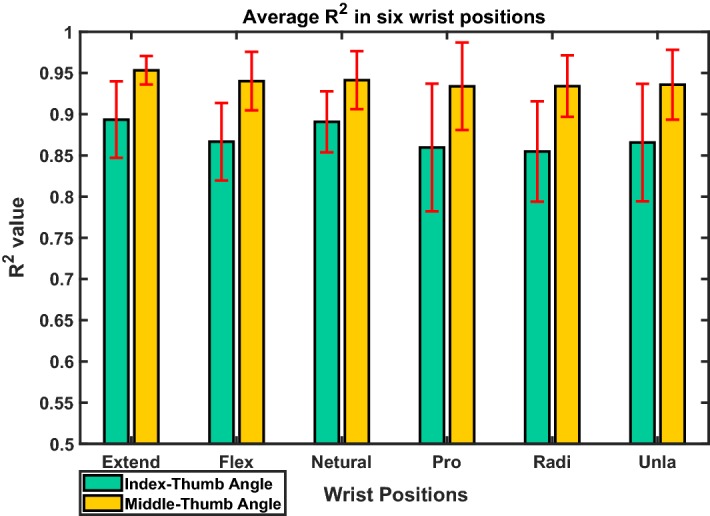
The average $R^2$ of two-step regression in six static wrist positions, the data are grouped to the classes based on the result of the classifier

Figure 11 shows the average $R^2$ of two-step regression in six static wrist positions. The data were grouped into the wrist positions based on the result of the classifier.

Two one-sided t-test (TOST) [33] to test the equivalence was run on the $R_{\theta _{TI}}^2$ and $R_{\theta _{TM}}^2$ to compare the two approaches. The value of level of significance was set to 0.05 ($\alpha = 0.05$), the lower limit of the equivalence interval was set to − 0.015 and the upper limit of the equivalence interval was set to 0.015. The null hypothesis was set as: $(\mu _{R^2_{one{\text{-}}step}}-\mu _{R^2_{two{\text{-}}step}})>0.015$ or $(\mu _{R^2_{one{\text{-}}step}}-\mu _{R^2_{two{\text{-}}step}})<-0.015$, and the alternative hypothesis was set as: $-0.015<(\mu _{R^2_{one{\text{-}}step}}-\mu _{R^2_{two{\text{-}}step}})<0.015$. The test resulted in $p < \alpha$ for both $R_{\theta _{TI}}^2$ and $R_{\theta _{TM}}^2$, which rejects the null hypothesis. Furthermore the confidence interval of the difference falls entirely inside the equivalence limits. This indicates the results from the two approaches are equivalent and they are not different from each other. The result indicates that the second approach had a similar performance to the first approach while it was more than 1.5 times slower than the one-step regression approach in training phase and significantly slower during the prediction of a new data point. The results suggest that one regression model for each angle ($\theta _{TI}$ and $\theta _{TM}$) can predict the angle value in the presence of variations of the position of the wrist. Subsequently, the first approach was selected to investigate the effect of the wrist position variation on the result.

### Analysis of the effect of the wrist position variation

All the possible combinations of removing one or more wrist positions from the training dataset were explored. Each combination was compared to the case of including all the wrist positions in the training dataset.

Table 5 indicate the result of including only one wrist position in the training dataset. As the result indicates, training on only one wrist position can notably decrease the $R^2$ value and increase the $RMSE\%$. In all cases for both $\theta _{TI}$ and $\theta _{TM}$ the statistical analysis for $R^2$ and $RMSE\%$ resulted in, $p \ll \alpha$. The p-value means we cannot conclude that there was no statistically significant difference between including all six positions in the training dataset or just including one wrist position. The result can confirm that including more than one wrist position in the training dataset is necessary, to predict continuous grasping movement in the presence of wrist position variations.

**Table 5:** The result of including only one wrist position in the training dataset, using one-step regression

Included wrist positions in training dataset	$\theta _{TI}$		$\theta _{TM}$	
	$R^2$	$RMSE\%$	$R^2$	$RMSE\%$
Extension, Flexion, Neutral, Pronation, Radial, Ulnar	0.872 ± 0.034	9.402 ± 1.052	0.941 ± 0.021	6.133 ± 0.843
Extension	0.412 ± 0.230	19.90 ± 2.79	0.566 ± 0.304	15.740 ± 4.460
Flexion	0.553 ± 0.345	22.64 ± 4.16	0.436 ± 0.339	18.359 ± 4.007
Neutral	0.550 ± 0.214	20.70 ± 3.50	0.593 ± 0.163	16.067 ± 2.818
Pronation	0.372 ± 0.160	17.56 ± 2.73	0.700 ± 0.170	13.533 ± 2.924
Radial	0.231 ± 0.157	17.45 ± 2.60	0.631 ± 0.203	15.027 ± 3.305
Ulnar	0.412 ± 0.277	18.10 ± 3.66	0.641 ± 0.277	14.120 ± 3.550

After that by comparing all different options for removing wrist positions from the training dataset, the positions that can be removed, without significant influence on the result of the prediction are identified. In case of removing one of the wrist positions from the training dataset, four options resulted in a p-value of more than 0.05. The $p > 0.05$ indicates that no statistically significant difference can be established between that specific option and including all six wrist position in the training dataset.

Table 6 presents the result of the wrist positions that need to be included in the training dataset and their corresponding $R^2$ and $RMSE\%$ value. The results indicated that the Extension, Pronation, Radial, or Ulnar wrist position could be removed from the training dataset. By removing any of these four wrist positions, no statistically significant difference can be established between the calculated $R^2$ and $RMSE\%$ with the values calculated with including all the six positions in the training dataset. The full result of the statistical analysis of each angle, with $R^2$ and $RMSE\%$, is provided in supplementary material (Additional file 1), which is the pair-wise comparison between all the 62 combinations and including all six wrist position in the training dataset. In addition, a sample of the FMG signal in different hand movements and wrist positions is provided in the supplementary material. Additional file 2 indicates that despite notable differences between some wrist positions in a hand movement, for example Extension and Pronation wrist positions, there are similarities in others, like Pronation and Ulnar deviation.

**Table 6:** The result of the algorithm using five wrist positions in the training dataset, one-step regression

Included wrist position in training dataset	$\theta _{TI}$		$\theta _{TM}$	
	$R^2$	$RMSE\%$	$R^2$	$RMSE\%$
Extension, Flexion, Neutral, Pronation, Radial, Ulnar	0.872 ± 0.034	9.402 ± 1.052	0.941 ± 0.021	6.133 ± 0.843
Flexion, Neutral, Pronation, Radial, Ulnar	0.821 ± 0.056	11.072 ± 1.375	0.901 ± 0.051	7.810 ± 1.575
Extension, Flexion, Neutral, Radial, Ulnar	0.842 ± 0.046	10.432 ± 1.362	0.927 ± 0.034	6.748 ± 1.221
Extension, Flexion, Neutral, Pronation, Ulnar	0.845 ± 0.046	10.320 ± 1.343	0.925 ± 0.039	6.797 ± 1.187
Extension, Flexion, Neutral, Pronation, Radial	0.832 ± 0.051	10.722 ±1.238	0.919 ± 0.039	7.239 ± 1.070

## Discussion

The FMG signal was investigated for continuous grasping movements estimation, with ten able-bodied participants. Concerning the goal of the study, two different methods were explored. The first was using a regression algorithm to predict the grasping movements; and the second was using a classification prior to the regression algorithm and six regression models corresponding to the wrist positions. The angle between Index finger and Thumb ($\theta _{TI}$) and Middle finger and Thumb ($\theta _{TM}$) were selected as the measures of the grasping movement.

Based on the presented result the two approaches had similar performance. However, the second approach is slower than the first approach, during both training and prediction phases. One regression model for the $\theta _{TI}$ angle and a regression model for $\theta _{TM}$ were able to estimate the continues grasping movement with an average $R^2$ of 0.906 and $RMSE\%$ of 7.77%. Pan et al. [11] were able to predict continuous finger movement in static wrist positions using sEMG with an average $R^2$ about 0.8. They used a switching regime approach and trained different regression models for different wrist positions. In addition, Pan et al. [11] estimates the testing time for a new data point to be 53.011 ms. Our work shows that FMG can predict a new data point in approximately 0.076 ms during the first approach. The present study implies that the FMG signal demonstrates comparable performance to sEMG in the same application, while there is no need to have different models for different wrist positions, as long as the data from different wrist positions are provided during the training.

This study demonstrated a more comprehensive approach compared to the work of Kadkhodayan et al. [23], and took one step closer to a practical case, as the effect of the wrist variation was considered in this work. Adewuyi et al. [5] investigate the possibility of training the system on one wrist position and use it with variations in the position of the wrist, using sEMG signal. Their result indicated that the variation of the wrist position can have a negative effect on the performance of the model. Our result shows that the same effect can be observed in FMG signal. If the system was only trained on one wrist positing, it was not able to predict the continuous grasping movement, with wrist position variation, with an average $R^2$ more than 0.6. Our further investigations revealed that it is possible to include five wrist positions in the training dataset and include all six positions in the testing phase. In other words, it is possible to remove Extension, Pronation, Radial, or Ulnar wrist position from the training dataset, without any statistically significant effect on the performance of the model. This provides four options for the training dataset, that include five wrist positions instead of six wrist positions (Table 6, rows 2–5). The Tukey HSD test was run to do a pairwise analysis between the four options, which provided six combinations. The results showed $p > 0.05$ in all six combinations. The p-value indicated no statistically significant differences can be established between the four options. In conclusion, it is possible to use any of them without degrading the performance of the system.

With respect to one step regression, the RF algorithm was able to have comparable results to SVR, and NNR while it was faster. In addition to that, the time of the training using RF algorithm was not sensitive to the size of the dataset. As mentioned in “Motion Data Processing” the $\theta _{TM}$ included 12 states and $\theta _{TI}$ included 18 states. The time of the training using the RF algorithm was 1.49 and 0.95 min for $\theta _{TI}$ and $\theta _{TM}$ respectively, and the training time of SVR algorithm was 6.17 and 2.35 for the angles respectively. This training time indicated that in RF algorithm by increasing the size of the dataset the training time did not increase drastically. The training time using SVR algorithm for $\theta _{TI}$ is over 2.5 times more than the training time for $\theta _{TM}$ with fewer data points. Regarding the NNR algorithm, the training time of the $\theta _{TI}$ is about twice the training time of the $\theta _{TM}$. The training times indicated the sensitivity of the SVR and NNR models to the size of the training dataset. The result for $\theta _{TM}$ dataset also indicates that the RF regression was a good alternative for the other commonly used regression models for the goal of this paper.

The results indicated, independent of the approach, $\theta _{TI}$ has a lower $R^2$ and higher $RMSE\%$ than $\theta _{TM}$. This result can be justified by looking at the hand movements that are included in the dataset for each angle. As it was mentioned in the “Methods” section, since the Middle finger is not moving in the Index Finger-Thumb movement, this movement is not included in the dataset of $\theta _{TM}$. The Index Finger-Thumb movement is more challenging to predict than the other two hand movements. The FMG band is placed on the forearm near the wrist. The tendons and muscles responsible for the movement of fingers, Thumb, and wrist are passing from this location. Keeping three hand movements in mind, in the Index Finger-Thumb movement, only Index and Thumb were moving. This means only the tendons and muscles that are controlling the Thumb and Index fingers were affecting the sensors that collect FMG, in a fixed wrist position. While in Two Fingers-Thumb movements the tendons and muscles responsible for moving Middle finger were influencing the signal as well. In the Large Diameter grip, all the fingers were moving, and there were more changes in each sensor’s reading in this movement. Every one of the sensors in the band can provide information for machine learning algorithm. Since in the Two Finger-Thumb movement and Large Diameter grip more sensors are influenced, more information is provided for the machine learning algorithm. This results in a better performance in estimating the angle in these two hand movements. Despite the facts mentioned above, it is worth noticing that both methods were able to estimate $\theta _{TI}$ with a $R^2$ more than 0.8.

## Conclusion

In this study, FMG signal was introduced and explored to provide continuous grasping movement prediction toward controlling partial hand prosthesis. With having the importance of the effect of the wrist movement on the performance of the grasping movement prediction in mind, the effect of the wrist movement on the FMG signals performance during predicting continuous grasping movement was explored. To the authors’ best knowledge this is the first work that considers the effect of the wrist movement on the performance of the FMG during continuous grasping movement prediction.

Two approaches were defined and examined for predicting continuous grasping movement in the presence of different wrist positioning. The first approach was to use one regression model for grasping movement prediction, irrespective of hand movements and wrist positions. During the second approach first, a classifier determined the wrist position that participant was in and after that, a regression model, trained for that wrist position, was used to predict the measures of the grasping movement. The two approaches had a similar performance, while the first approach was faster than the second approach. This indicates one regression model with adequate training data was able to predict the grasping movement.

The effect of the variations in the wrist position was investigated. The result concluded that it is necessary to include at least five wrist positions in the training dataset to be able to predict continuous grasping movement in different wrist positions.

In short, the presented study indicates the potential of FMG signal to be used as a control technology to provide continuous grasping movement control for partial hand amputees. Future works can investigate the possibility of improving the performance by adding feature extraction from the FMG signal and studying deep learning algorithms for regression. Additionally, more grasping movements can be included in the study as the number of the grasping movements covered in this study were limited to three grasp movements. To continue the work in the future, it is essential to test the ability of the signal to perform grasping movement regression, while the wrist is not constrained to specific angles and include a continuous wrist movement estimation in the algorithm if it is needed. It is also necessary to test the signal to control a simulated hand or a robotic hand gripper in real-time. In addition, the effect of the FMG band placement on the limb as well as the effect of the fatigue on performance of the system should be investigated. Also, the effect of object handling and the resistance from holding and squeezing an object should be investigated. At last, it is important to test the result of the study with partial hand amputee participants and test the potential and limitations of the signal and the algorithm.

## Additional files

**Additional file 1.** Full results of the statistical analyses of the Effect of the wrist position variation. This file includes the result of pairwise analysis between all the 62 options for removing wrist positions from the training dataset and including all six positions in the training dataset. The analysis are done for θ_TI_ and θ_TM_ independently. For each angle the analysis are done on *R*^2^ and *RMSE%*.

**Additional file 2.** Snap shot of hand movement signal in different wrist positions. This file includes a graph that shows the FMG signal in different hand movements and wrist positions. The graph shows a sample repetition for each movement in each wrist position form one of the subjects.

## Abbreviations

FMG: force myography
*R*^2^: correlation of determination
RF: random forest
sEMG: surface electromyography
MCP: metacarpophalangeal
PIP: proximal interphalangeal
FSR: force sensing resistor
PTF: polymer thick film
IP: interphalangeal
LR: linear regression
SVR: support vector regression
NNR: neural network regression
LDA: linear discriminate analysis
$RMSE\%$: percentage root mean square error
SVM: support vector machine

## Footnotes

1 https://vincentsystems.de/en/; https://www.touchbionics.com/
