# Supplementary material for: Regressing grasping using force myography: an exploratory study
Source: Biomed Eng Online. 2018 Oct 23;17:159. doi: 10.1186/s12938-018-0593-2 (PMC6199756; doi:10.1186/s12938-018-0593-2)
Supplement: Supplementary file 1 — Additional file 1. Full results of the statistical analyses of the Effect of the wrist position variation. This file includes the result of pairwise analysis between all the 62 options for removing wrist positions from the training dataset and including all six positions in the training dataset. The analysis are done for θTI and θTM independently. For each angle the analysis are done on R2 and RMSE%. [file 12938_2018_593_MOESM1_ESM.pdf]

Additional File2 – Snap Shot of Hand Movement Signal in Different Wrist Positions

This file includes a sample of the FMG signal in different hand movements and wrist positions. The graphs from top to bottom show the opposed Thumb-Index finger grip, opposed Thumb-Two finger grip, and oppose Heavy Wrap. The sections of the graphs that are number as 1-6 indicate the Extension wrist position, Flexion wrist position, Neutral wrist position, Pronation wrist position, Radial wrist position and Ulnar wrist position. Different colors on the graph indicate the different sensor values.

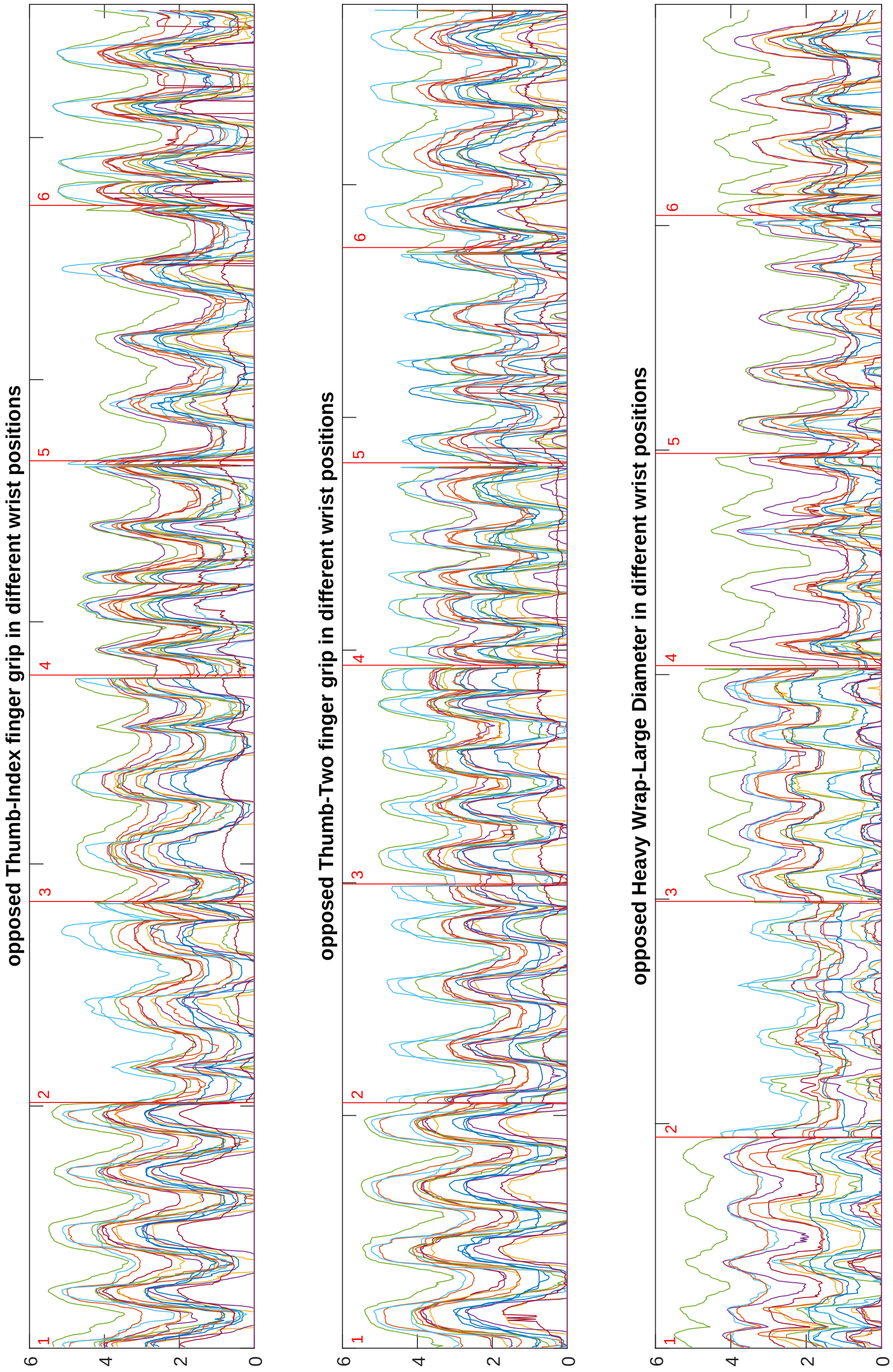

Figure 1 Comparison between the FMG signal in different wrist positions
