# Supplementary material for: Regressing grasping using force myography: an exploratory study
Source: Biomed Eng Online. 2018 Oct 23;17:159. doi: 10.1186/s12938-018-0593-2 (PMC6199756; doi:10.1186/s12938-018-0593-2)
Supplement: Supplementary file 2 — Additional file 2. Snap shot of hand movement signal in different wrist positions. This file includes a graph that shows the FMG signal in different hand movements and wrist positions. The graph shows a sample repetition for each movement in each wrist position form one of the subjects. [file 12938_2018_593_MOESM2_ESM.pdf]

## Additional File1 – Full Results of the statistical analyses of the Effect of the Wrist Position variation

Each option for removing one or more wrist positions is identified with a group number. The case of including all wrist positions in the training data set is identified as "group 1". Figures 1 - 4 indicate the pairwise comparison of the different options for training data set. The figures can indicate the overlapping options. We can not establish any statistically significant difference between the options that have an overlapping interval. Table 1 indicates the removed wrist positions and their group id.

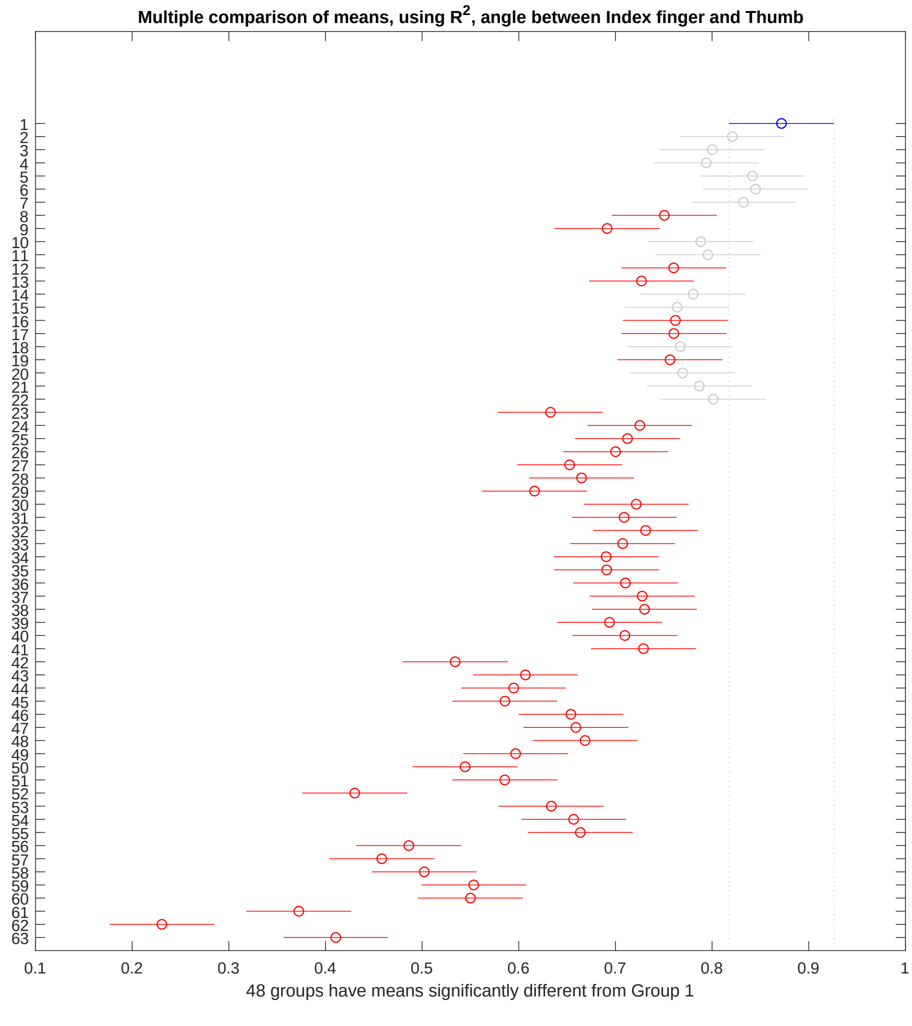


**Figure 1** The multiple comparison of mean, using $R^2$, $\theta_{TI}$

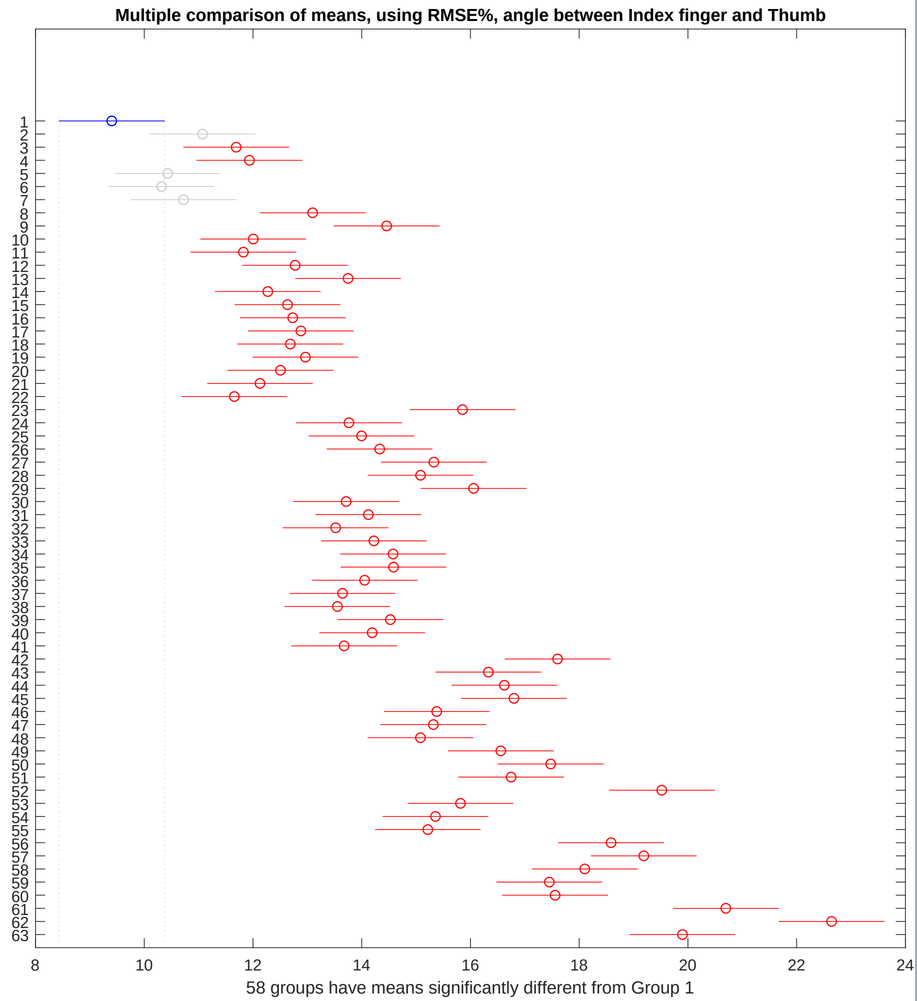


**Figure 2** The multiple comparison of mean, using $RMSE\%$, $\theta_{TI}$

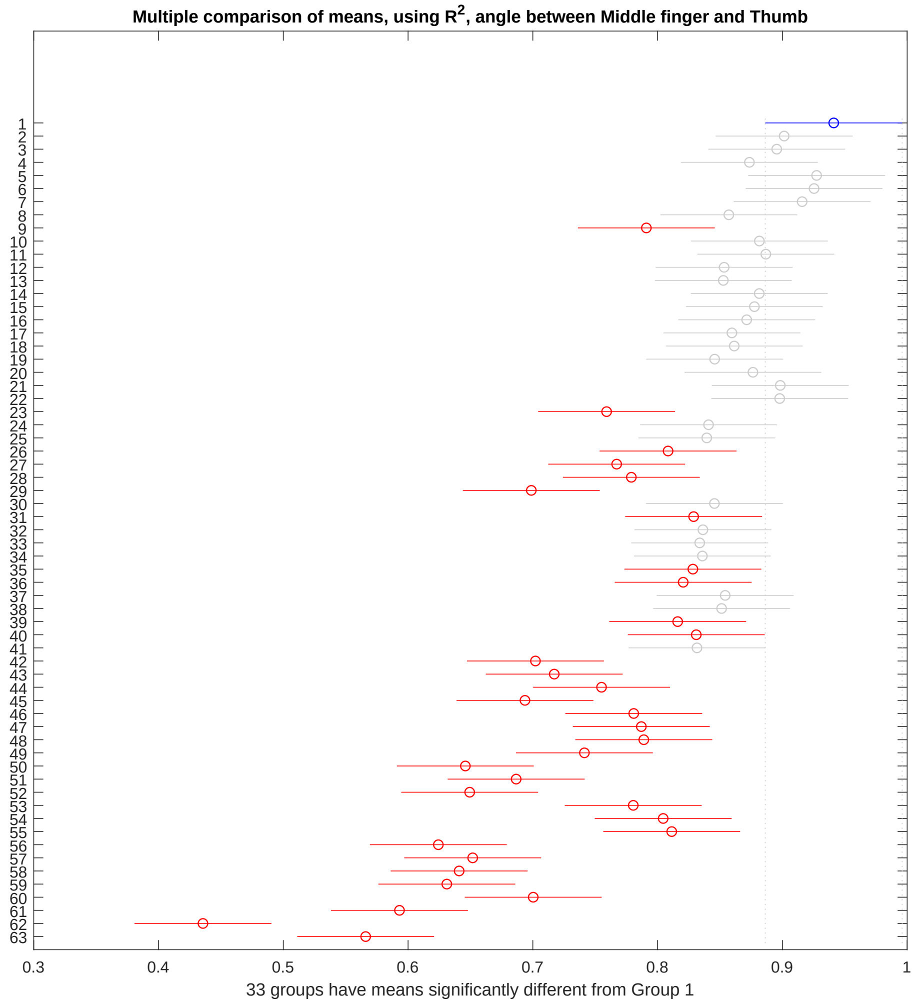


**Figure 3** The multiple comparison of mean, using $R^2$, $\theta_{TM}$

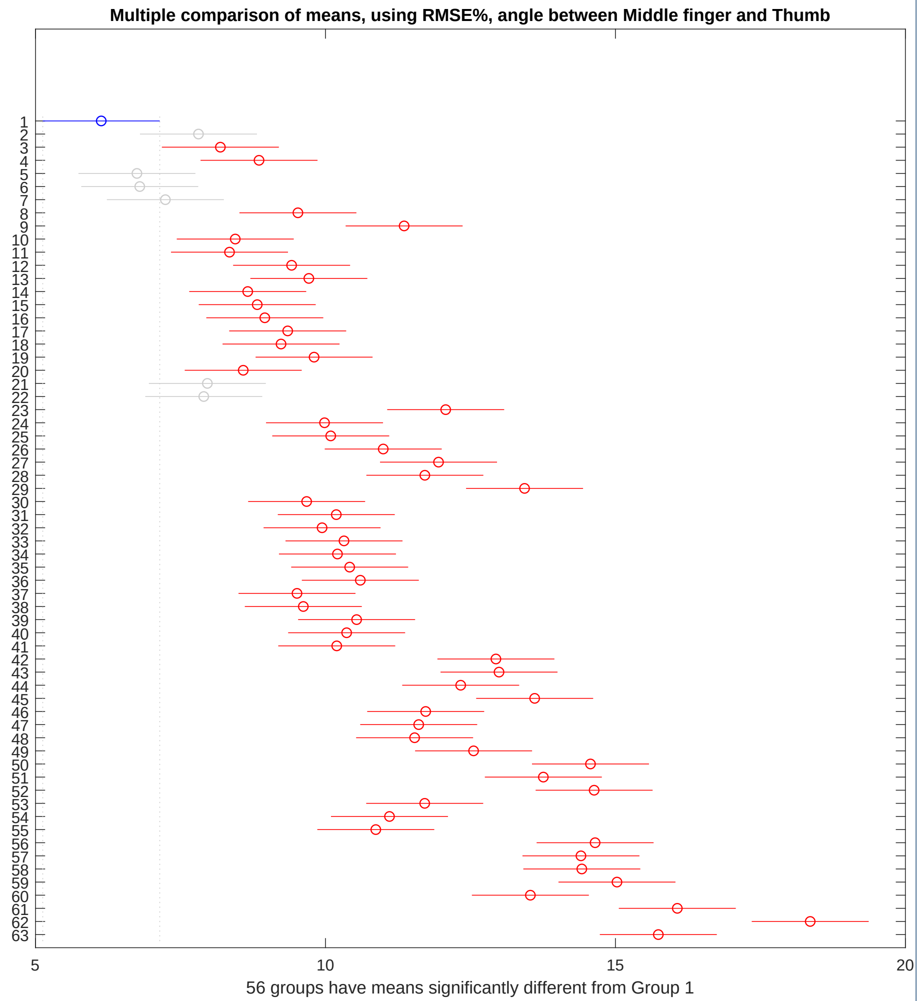


**Figure 4** The multiple comparison of mean, using $RMSE\%$, $\theta_{TM}$

**Table 1** Removed wrist positions and their group id

| group id | removed wrist positions | group id | removed wrist positions | group id | removed wrist positions |
|---|---|---|---|---|---|
| 1 | None | 22 | Radial, Ulnar | 43 | Extension, Flexion, Neutral, Pronation |
| 2 | Extension | 23 | Extension, Flexion, Neutral | 44 | Extension, Flexion, Neutral, Radial |
| 3 | Flexion | 24 | Extension, Flexion, Pronation | 45 | Extension, Flexion, Neutral, Ulnar |
| 4 | Neutral | 25 | Extension, Flexion, Radial | 46 | Extension, Flexion, Pronation, Radial |
| 5 | Pronation | 26 | Extension, Flexion, Ulnar | 47 | Extension, Flexion, Pronation, Ulnar |
| 6 | Radial | 27 | Extension, Neutral, Pronation | 48 | Extension, Flexion, Radial, Ulnar |
| 7 | Ulnar | 28 | Extension, Neutral, Radial | 49 | Extension, Neutral, Pronation, Radial |
| 8 | Extension, Flexion | 29 | Extension, Neutral, Ulnar | 50 | Extension, Neutral, Pronation, Ulnar |
| 9 | Extension, Neutral | 30 | Extension, Pronation, Radial | 51 | Extension, Neutral, Radial, Ulnar |
| 10 | Extension, Pronation | 31 | Extension, Pronation, Ulnar | 52 | Extension, Pronation, Radial, Ulnar |
| 11 | Extension, Radial | 32 | Extension, Radial, Ulnar | 53 | Flexion, Neutral, Pronation, Radial |
| 12 | Extension, Ulnar | 33 | Flexion, Neutral, Pronation | 54 | Flexion, Neutral, Pronation, Ulnar |
| 13 | Flexion, Neutral | 34 | Flexion, Neutral, Radial | 55 | Flexion, Neutral, Radial, Ulnar |
| 14 | Flexion, Pronation | 35 | Flexion, Neutral, Ulnar | 56 | Flexion, Pronation, Radial, Ulnar |
| 15 | Flexion, Radial | 36 | Flexion, Pronation, Radial | 57 | Neutral, Pronation, Radial, Ulnar |
| 16 | Flexion, Ulnar | 37 | Flexion, Pronation, Ulnar | 58 | Extension, Flexion, Neutral, Pronation |
| 17 | Neutral, Pronation | 38 | Flexion, Radial, Ulnar | 59 | Extension, Flexion, Neutral, Pronation |
| 18 | Neutral, Radial | 39 | Neutral, Pronation, Radial | 60 | Extension, Flexion, Neutral, Radial, Ulnar |
| 19 | Neutral, Ulnar | 40 | Neutral, Pronation, Ulnar | 61 | Extension, Flexion, Pronation, Radial, Ulnar |
| 20 | Pronation, Radial | 41 | Neutral, Radial, Ulnar | 62 | Extension, Neutral, Pronation, Radial, Ulnar |
| 21 | Pronation, Ulnar | 42 | Pronation, Radial, Ulnar | 63 | Flexion, Neutral, Pronation, Radial, Ulnar |
